# A BAC-based physical map of the Nile tilapia genome

**DOI:** 10.1186/1471-2164-6-89

**Published:** 2005-06-09

**Authors:** Takayuki Katagiri, Celeste Kidd, Elizabeth Tomasino, Jesse T Davis, Cassandra Wishon, Justin E Stern, Karen L Carleton, Aimee E Howe, Thomas D Kocher

**Affiliations:** 1 Hubbard Center for Genome Studies, University of New Hampshire, Durham, New Hampshire 03824, USA; 2 Laboratory of Fish Health Management, Tokyo University of Marine Science and Technology, Tokyo, Japan; 3 Department of Food Science & Technology, Cornell University, Ithaca, New York USA

## Abstract

**Background:**

Cichlid fishes, particularly tilapias, are an important source of animal protein in tropical countries around the world. To support selective breeding of these species we are constructing genetic and physical maps of the tilapia genome. Physical maps linking collections of BAC clones are a critical resource for both positional cloning and assembly of whole genome sequences.

**Results:**

We constructed a genome-wide physical map of the tilapia genome by restriction fingerprinting 35,245 bacterial artificial chromosome (BAC) clones using high-resolution capillary polyacrylamide gel electrophoresis. The map consists of 3,621 contigs and is estimated to span 1.752 Gb in physical length. An independent analysis of the marker content of four contigs demonstrates the reliability of the assembly.

**Conclusion:**

This physical map is a powerful tool for accelerating genomic studies in cichlid fishes, including comparative mapping among fish species, long-range assembly of genomic shotgun sequences, and the positional cloning of genes underlying important phenotypic traits. The tilapia BAC fingerprint database is freely available at .

## Background

The family Cichlidae is one of the most species-rich families of vertebrates [1]. More than 3,000 species of cichlid fishes are distributed from Central and South America, through Africa and Madagascar, to southern India [2]. Although cichlids are diverse and dominant components of the freshwater fauna of both the Old and New Worlds, it is in the lakes of East Africa that they have undergone the spectacular adaptive radiations for which the group is best known [3]. Cichlids are an emerging model system for studying a broad range of questions at the interface of organismal biology and genomics [4].

Tilapias (*Oreochromis *spp.) are cichlid fishes which have become one of the most important species in global aquaculture. Native to Africa, several species of tilapia have been introduced to tropical areas of Asia and the Americas to increase supplies of animal protein. World aquaculture production of tilapia is second only to carp, and now exceeds 1.5 million tons per year [5].

The Nile tilapia (*Oreochromis niloticus*) genome contains 1.06 gigabase pairs distributed over 22 chromosome pairs [6]. Several partial genetic linkage maps of tilapia have been produced [7-9]. The latest and most complete map orders 550 loci in 24 linkage groups spanning a total of 1311 cM [10].

Here we present a physical map of the tilapia genome based on restriction fingerprints of more than 35,000 large-insert bacterial artificial chromosome (BAC) clones. This physical map will help speed positional cloning in tilapia, and will facilitate the long-range assembly of a tilapia genome sequence.

## Results and discussion

### BAC fingerprinting

**Figure 1:**
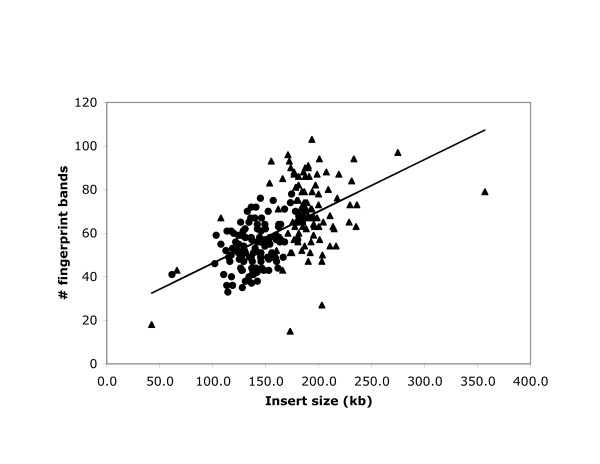
**Relationship between number of fingerprint bands and clone insert size. **Clones from the T3 library shown as circles, T4 library shown as triangles. The line shows the regression: number of bands = 22.37 + 0.238 * insert size (kb).

We processed 40,704 clones from libraries 3 and 4, and obtained valid fingerprints from a total of 35,245 clones (87% success; Tables 1 and 2). Library 3 has an average insert size of 145 kb, and produced an average of 53.9 valid bands per clone. Library 4 has an average insert size of 194 kb, and produced an average of 69.8 bands per clone. Figure 1 shows the regression of fingerprint band number on clone insert size. Together, the fingerprinted clones represent an estimated 5.6-fold coverage of the tilapia genome.

**Table 1:** BAC libraries fingerprinted for the tilapia physical map. Construction of these BAC libraries is described in Katagiri *et al. *[16]. Copies of the libraries are available as plates and filters from .

**Library**	**Cloning site**	**Vector**	**Mean insert size (kb)**	**No. of clones fingerprinted**	**Valid bands per clone**	**Genome coverage**
HCGS-03TI	HindIII	pBAC-lac	145	18,700	53.9	2.56
HCGS-04TI	HindIII	pBAC-lac	194	16,545	69.8	3.02
	Total		182	35,245	61.4	5.58

**Table 2:** Summary of the tilapia physical map

**Number of clones processed**	**40,704**
T3 library	20,736
T4 library	19,968
	
**Number of clones used for contig assembly**	**35,245**
T3 library	18,700
T4 library	16,545
Average success rate	87%
	
**Number of singletons**	**2,647**
	
**Number of contigs**	**3,621**
2–4 clones	1,646
5–5 clones	973
10–25 clones	771
26–50 clones	188
51–100 clones	34
101–200 clones	8
>200 clones	1
	
**Physical length of the contigs**	**1.752 Mb**

### Contig assembly

Contigs were assembled from the fingerprint data using the computer program FPC version 6.0 [11,12]. We estimated the sizing accuracy of the capillary sequencer by analyzing the size of the vector band in 200 clones. The mean size was 246.20, with a standard deviation of ± 0.253 bp. We therefore multiplied all fragments sizes by 10, and used a fixed tolerance of 5, corresponding to 0.5 bp, in the FPC analysis.

**Figure 2:**
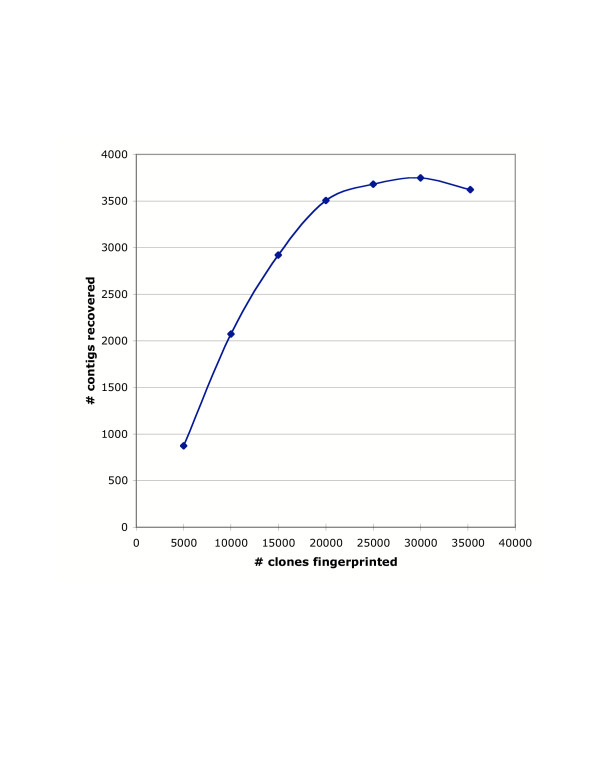
**Coalescence of contigs during the fingerprinting process. **The number of contigs rises to a maximum of 3,748 contigs after fingerprinting 30,000 clones. With additional fingerprinting, it appears that the contigs are beginning to coalesce. All analyses performed with a tolerance of 5 and cutoff threshold of 1e-08.

Using a cutoff stringency of 1e-08, the number of contigs reached a plateau of approximately 3,500 after 20,000 clones had been fingerprinted. The number of contigs reached a maximum of 3,748 contigs at 30,000 clones, and dropped to 3,621 contigs in the final analysis of 35,234 clones (Fig 2). A total of 32,598 clones (92.5%) were placed in contigs. Only 2,647 clones remained as singletons (Table 2).

The contigs contain an average of 9.0 clones each, and had an average estimated length of 389.9 kb. The assembled contigs have an estimated length of 1.752Gb, or about 1.65x the genome length. Half of the total assembly length is in the largest 1,054 contigs. The top half of the contigs (1,630 clones) contained 69% of the total length of the assembly.

### Contig reliability

We used several different approaches to assess contig reliability. The first was to determine the stability of contigs at different cutoff values. Increasing the stringency of assembly, from 1e-08 to 1e-09, increased the number of contigs from 3,621 to 4,008. This means that approximately 200 contigs were split at the higher stringency, which is less than 5% of initial total.

**Figure 3:**
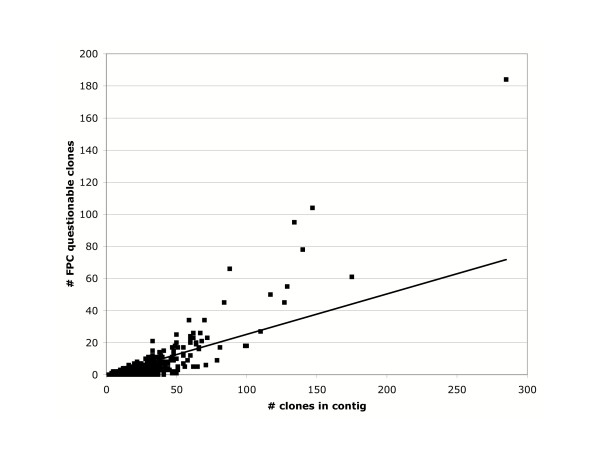
**Q scores for contigs of different size. **The number of questionable clones identified by FPC rises with the size of the contig. Very large contigs tend to have a disproportionate number of Q's, suggesting improper assembly of repetitive sequences. The line represents a least squares fit of y = 0.252x (r^2 ^= 0.54).

FPC identified a total of 3,127 questionable clones (Q's) in the assembly, an average of 0.86 Q's per contig. However, the distribution was strongly skewed from Poisson expectations. 2,891 contigs (92.5%) had no Q's called. Most of the questionable clones were in a few large contigs (Table 3). More than half of the Q's were in the 58 contigs with 10 or more questionable clones. Since the number of Q's was strongly correlated with the number of clones in the contig (Fig 3), we suspect this represents improper assembly of clones containing repetitive sequences.

**Table 3:** Distribution of FPC questionable clones (Q's). Poisson expectations calculated from the average of 0.86 Q's per contig.

**#Q's**	**# Contigs**	**Poisson**
0	2891	1526
1	328	1318
2	133	569
3	93	163
4	42	35
5	24	6
6	14	1
7	22	0
8	10	0
9	6	0
10	5	0
11	8	0
12	4	0
13	2	0
14	1	0
15	3	0
16	1	0
17	5	0
18	3	0
19	1	0
20+	25	0

Cichlid fishes have an expanded set of opsin genes relative to tetrapods. Changes in the expression of these genes are responsible for differences in visual sensitivity among species [13]. In order to identify the regulatory regions for these genes, we isolated BAC contigs containing opsin genes. PCR screening of pooled BAC DNAs identified clones containing the SWS1, RH2 and LWS genes. The FPC database was then used to identify overlapping BACs at a tolerance of 5 and cutoff threshold of 1e-08. The SWS1 contig contained six clones, all of which were positive for the SWS gene by PCR. The RH2 contig contained 18 clones, 11 of which were positive for the RH2 gene. Probes derived from end sequencing of these BACs were used to verify that the remaining 7 clones were members of a genuine contig. The LWS contig contained 10 clones, 5 of which contained the LWS gene. Probes developed from the end sequences of these clones verified that four of the remaining five clones were members of an overlapping contig. The fifth clone should not have been included in this contig. The genes in this contig are homologous to the SWS2 and LWS genes located on scaffold 5 of Fugu assembly version 3.0. Two of the six BAC end sequences derived from the tilapia contig had BLAST hits to Fugu scaffold 5, providing strong evidence for homology of this contig to a 100 kb region of the Fugu genome.

**Figure 4:**
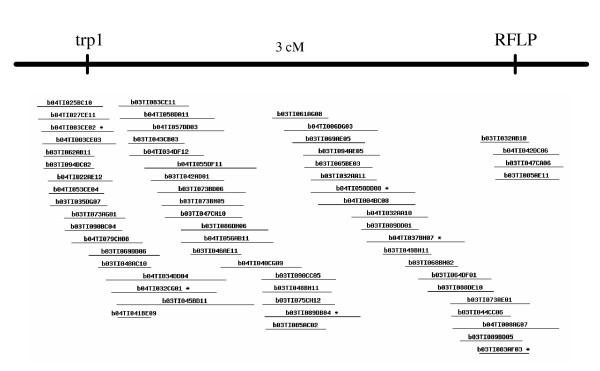
**Contig containing the *trp1 *gene. **PCR screening identified *trp1 *sequences in BAC clone b03TI073AG01, near one end of this contig. A RFLP was developed by shotgun sequencing of clone b04TI008AG07, near the other end of the contig. Genetic mapping shows these markers are about 3 cM apart, confirming the utility of this contig spanning approximately 2 Mb.

Finally, in the course of positional cloning a mutation for red body color in tilapia, we identified a BAC containing the tyrosinase-related protein 1 (*trp1*) gene. Using a reduced FPC stringency (tol = 5, 1e-06) this BAC was near one end of a contig of 70 clones which is estimated to span 1.97 Mb. An RFLP was identified from a clone at the opposite end of this contig, and was mapped 3cM from *trp1 *in a large F2 progeny (Fig 4). This result emphasizes the utility of the fingerprint database, even at reduced stringencies of assembly.

## Conclusion

East African cichlid fishes, including the tilapias as well as the closely related and highly diverse haplochromine cichlids, constitute more than 5% of vertebrate species. An international consortium has come together to develop genomic tools for studying these fishes . Resources already developed include a genetic map with more than 550 microsatellite markers [10], and a collection of more than 50,000 ESTs [14,15].

The physical map described in this paper is a further step in building the infrastructure to support complete sequencing of the cichlid genome. Fingerprinting of additional clones from these libraries would undoubtedly allow further coalescence of contigs, but it is not clear how cost-effective this approach would be. The current set of 3500 contigs is a manageable number for anchoring to physical and comparative maps. A logical next step in this research would be analysis of the gene content of these contigs to relate the contigs to the sequences of other fish genomes. In the meantime, the physical map will facilitate the positional cloning of genes controlling economically important traits in tilapia, as well as the genes underlying the spectacular adaptive radiation of cichlids in the lakes of East Africa.

## Methods

### Source BAC libraries

Four BAC libraries have been constructed for *Oreochromis niloticus *[16]. All four libraries were constructed from the sperm of a single male (#00-0135-EA1B) from a strain originating from Lake Manzallah, Egypt and maintained at the University of Stirling, UK. We fingerprinted clones from the two libraries with the largest average insert size (Table 1). Insert sizes of 200 BACs from each library were determined by NotI digestion and comparison to a lambda PFG ladder (New England Biolabs, Beverly MA). Plates and filters of these clones are available on a cost-recovery basis from the Hubbard Center for Genome Studies .

### BAC fingerprinting

BAC DNA was isolated using a modified alkaline lysis method [17]. Briefly, BAC clones were inoculated into 96-deep well plates. Each well contained 1.5 ml of 1x LB media with chloramphenicol at a concentration of 12.5 μg/ml. The plates were covered with Qiagen Airpore tape sheets (Cat# 19571) and incubated at 37°C for 20–21 hours on a Bellco mini-orbital shaker. Restriction fingerprints were obtained following the approach of Ding et al. [18]. The DNA was double-digested with HindIII and HaeIII and the HindIII ends labelled with fluorescently labelled ddGTP in a fill-in reaction using the reagents from a Beckman DTCS sequencing kit. The fragments were sized on Beckman CEQ2000 capillary DNA sequencers using the CEQ-600 molecular weight standard (Beckman Coulter, Fullerton CA).

### BAC contig assembly

Every chromatogram was manually reviewed to confirm the peaks identified by the Beckman CEQ8000 software. Only the bands between 80 to 620 bp were used for contig assembly. The chromatograms and associated peak values were then stored in a MySQL database for further analysis. Contig assembly was done using the computer program FPC (vers. 6.0; ) [12]. The resulting contigs are displayed using a new www-based viewer which mimics the WebFPC interface . This viewer is written in PHP and generates html in response to queries of the database.

### DNA markers and BAC library screening

To facilitate screening of the BAC libraries by PCR we constructed pools of the bacterial cultures. The pools were constructed from 252 96-well plates (144 from library T3 and 108 from library T4). This is equivalent to 2x coverage, or 2 Gb equivalents, from each library. We collected row and column pools from each plate using a Beckman Biomek2000 robotic pipettor. The row pools from each plate were pooled by hand to produce 252 plate pools. The plate pools were grouped into one of 10 arrays of either 4 × 6 or 5 × 6 plates. We then constructed pools from the rows and columns in each of these arrays. Finally, we constructed 10 superpools corresponding to the groups of plates in each array. This allowed us to identify positive clones by PCR in a sequence of 3 experiments. We first attempted amplification from each of the 10 superpools. We then analyzed the row and column plate pools for each positive superpool to identify the plate. Finally, we analyzed the 8 row and 12 column pools from each positive plate to identify the clone.

## Authors' contributions

TK developed the DNA extraction and fingerprinting protocols. CK, ET, JTD and CW obtained the fingerprints and imposed quality controls on data entering the analysis pipeline. JES developed the databases and software interfaces for displaying the FPC results on the www. KLC and AEH tested contig reliability by sequencing and probe hybridization. TDK conceived the project, supervised its execution and wrote the manuscript.
